# Real-world effectiveness and safety of esketamine intranasal spray combined with treatment-as-usual in psychiatric inpatients

**DOI:** 10.1192/j.eurpsy.2024.539

**Published:** 2024-08-27

**Authors:** E. Kavakbasi, M. Yilmaz, Ö. Bulut, H. Berndt, B. T. Baune

**Affiliations:** ^1^Department of Psychiatry, University Hospital Münster, University of Münster, Münster, Germany; ^2^Department of Psychiatry, Melbourne Medical School, The University of Melbourne, Melbourne; ^3^The Florey Institute of Neuroscience and Mental Health, The University of Melbourne, Parkville, Australia

## Abstract

**Introduction:**

Esketamine intranasal spray has been approved in both the USA and EU as a novel treatment in patients with treatment-resistant major depression (TRD) and for the management of acute depressive emergencies during the course of major depressive disorder (MDD). Real-world data on the effectiveness and safety of esketamine nasal spray in clinical use are limited.

**Objectives:**

To investigate the clinical effects and safety of esketamine nasal spray on depression severity and suicidal ideation during inpatient treatment in n=76 patients in a German university hospital.

**Methods:**

In this retrospective chart review, we analyzed the change in depression severity and safety after a treatment series with esketamine nasal spray combined with treatment-as-usual in patients with treatment-resistant depression (TRD) in inpatient treatment setting of a University Hospital. Depression severity has been rated with the Montgomery–Åsberg Depression Rating Scale (MADRS) as well as with the BDI-II (Beck Depression Inventory-Second Edition) before and after the treatment series. The intensity of suicidal ideation has been evaluated using MADRS item 10 on suicidal thoughts.

**Results:**

A total of 76 patients have been included (women 55.3, n=42) in this analysis. Mean BDI-II pre-treatment was 37.6 and mean MADRS was 33.6 corresponding to severe depression. Mean score on item-10 pre-treatment was 2.4 (median 2.0). On average patients received 10.9 sessions (standard deviation 4.2, median 11.0) of esketamine nasal spray (min 1, max. 19 sessions). There was clear improvement after the treatment series in both the BDI-II (mean change -10.1, p < 0.001) as well as in MADRS score (mean reduction -10.0, p < 0.001). Suicidal ideation on item-10 also decreased significantly (-0.9, p < 0.001). The effect sizes were large for all three measures: Cohen’s d 1.050 for BDI-II; 0.986 for MADRS and 0.742 for changes in suicidal ideation. Overall, esketamine treatment was well tolerated. In five cases esketamine treatment has been terminated early (after a mean of 3.4 sessions) due to dissociations (n=4; 5.3%) or due to non-response (n=1).

**Conclusions:**

Esketamine nasal spray is a novel effective and safe treatment option, which leads to significant decrease in depression severity as well as in suicidal ideation. More data from real-world patients are needed to position esketamine in the algorithm of depression treatment. Rate of treatment discontinuation due to side-effects in this study was comparable to those in other esketamine studies (4.2% in Reif et al, NEJM, 2023).

**Disclosure of Interest:**

None Declared

